# FPIES in exclusively breastfed infants: two case reports and review of the literature

**DOI:** 10.1186/s13052-020-00910-8

**Published:** 2020-10-06

**Authors:** Francesco Baldo, Martina Bevacqua, Cristiana Corrado, Daniela Nisticò, Laura Cesca, Valentina Declich, Roberto Dall’Amico, Egidio Barbi

**Affiliations:** 1 Department of Medicine, Surgery and Health Sciences, University of Trieste, Piazzale Europa 1, 34127 Trieste, Italy; 2 Department of Pediatrics, AAS n.5 Friuli Occidentale, via Montereale 24, 33170 Pordenone, Italy; 3 Department of Pediatrics, Institute for Maternal and Child Health, IRCCS Burlo Garofolo, via dell’Istria 65/1, 34137 Trieste, Italy

**Keywords:** FPIES, Food protein-induced Enterocolitis syndrome, Breastfeeding, Breast milk, Methemoglobinemia

## Abstract

**Background:**

Food Protein-Induced Enterocolitis Syndrome (FPIES) is a non IgE-mediated food allergy that generally affects children in the first year of life. Usually symptoms break out when formula milk or solid foods are introduced for the first time but they might also appear in exclusively breastfed infants, since the trigger elements, especially cow’s milk proteins, can be conveyed by maternal milk as well. FPIES in exclusively breastfed babies is a very rare clinical condition and only few cases have been reported in the medical literature.

**Case presentation:**

We describe two cases of FPIES in exclusively breastfed babies. The first one is a two-month-old infant with a brief history of vomit and diarrhea that presented to the Emergency Department in septic-like conditions. The main laboratory finding was a significant increase in methemoglobin (13%). Clinically, we noted that, when breastfeeding was suspended, diarrhea drastically improved, and vice versa when maternal milk was reintroduced. An amino acid-based formula allowed a complete normalization of the symptoms. The second one is a three-month-old infant admitted for a 3 days history of persistent vomit and diarrhea. Blood tests showed a raised level of methemoglobin (7%). An esophagogastroduodenoscopy was performed and biopsies showed an eosinophilic infiltration of the duodenal mucosa. A maternal exclusion diet and an amino acid-based formula allowed a rapid regularization of the bowel function.

**Conclusions:**

We searched all the cases of FPIES in exclusively breastfed babies reported in the medical literature, identifying eight patients, with an average age of 3 months (range 15 days – 6 months). The majority of the cases were initially diagnosed as gastroenteritis or sepsis, five cases were characterized by an acute on chronic scenario and cow’s milk was the most frequently involved food. Methemoglobin was never tested. An oral food challenge test was performed in two patients.

FPIES in exclusively breastfed infants is a rare condition that, in the presence of compatible history and symptoms, should be considered also in exclusively breastfed babies. The evaluation of methemoglobin can simplify the diagnostic process.

## Background

Food protein-induced enterocolitis syndrome (FPIES) is a non IgE-mediated food allergy that generally affects patients in the first year of life [1–10]. It is caused by a reaction against food proteins in the gut, that determines an increased permeability of the intestines and a fluid shift into the gut lumen, leading to vomit, diarrhea and eventually distributive shock. Patients with FPIES are reactive to one food in 65–80% of the cases, while two or more foods are involved in the remaining cases. Cow’s milk is the most frequent trigger, followed by soy, grains and rice. FPIES is distinguished into an acute and a chronic form, based on the timing and duration of the symptoms (Table 1).

**Table 1:** Symptoms and laboratory values of chronic and acute FPIES

Chronic FPIES	Acute FPIES
**Symptoms**	
Recurrent vomiting	Unstoppable vomiting (1 to 3 h after meal)
Chronic diarrhea	Diarrhea (with/without blood) 4–6 h after meal
Dehydration	Lethargy (hypotension, hypothermia)
Abdominal distension	Pallor
Weight loss, poor growth	Abdominal distension
**Laboratory values**	
Anemia	Neutrophilia
Hypoalbuminemia	Thrombocytosis
Neutrophilia/eosinophilia	Metabolic acidosis
Metabolic acidosis	Methemoglobinemia
Methemoglobinemia	Stool leukocytes and eosinophils
Reducing substances in stools	Blood in stools
	Reducing substances in stools

Usually symptoms break out when formula milk or solid foods are introduced for the first time. However, as reported by Mehr and colleagues, they might also appear in exclusively breastfed infants in less than 5% of the cases [2]. As a matter of fact, pathogenic proteins can be conveyed by maternal milk as well [11–13]. For example, Zhu et al. observed that peptides and proteins originating from bovine milk products were the dominant nonhuman proteins observed in human milk, notably bovine caseins (α-S1-, α-S2-, β-, κ-caseins) and β-lactoglobulin [14]. Although these elements are supposed to play a role in allergy prevention, in rarer cases they were reported to cause IgE and non IgE-related food reactions [14].

While the International Consensus Guidelines for the Diagnosis and Management of FPIES established the criteria for the diagnosis of acute FPIES (Table 2), there are still no recognized criteria for the chronic form [9]. The presumptive diagnosis of chronic FPIES is based on the resolution of its clinical manifestation in several days or weeks from the avoidance of the trigger food and the acute recurrence of symptoms at the reintroduction of it. Oral food challenge (OFC) is the gold standard to make the diagnosis in the presence of an unclear history.

**Table 2:** Diagnostic criteria of acute FPIES (from reference [1], modified)

The diagnosis of acute FPIES requires the presence of vomiting (1–4 h after the ingestion of the suspected food) and the absence of classic IgE mediated allergic skin respiratory symptoms **(major criteria)**, plus at least three of the following **minor criteria:**	
A second (or more) episode of repetitive vomiting after eating the same suspected food	
Extreme lethargy with any suspected reaction	
Marked pallor with any suspected reaction	
Need for Emergency Department visit with any suspected reaction	
Need for intravenous fluid support with any suspected reaction	
Hypotension	
Hypothermia	
If only a single FPIES reaction has occurred, it is strongly recommended to perform a diagnostic food challenge	

FPIES can mimic other medical conditions such as gastroenteritis, sepsis, intussusception, or inborn errors of metabolism [15] (Table 3). Lee et al., in a retrospective case-control study, underlined the difference between FPIES, gastroenteritis, and sepsis in young children who showed up the Emergency Department with vomit. They suggest that the absence of fever, lethargy, floppiness, pallor, and normal C-reactive protein are more indicative of FPIES, while fever, diarrhea, and elevated C-reactive protein are features of other conditions [16]. However, a clear distinction between these conditions might be difficult. For example, as reported by Kimura et al., in a cohort of patients with FPIES, fever and raised C reactive protein levels are present in about a third of them [17].

**Table 3:** Differential diagnosis of FPIES (from reference [1], modified)

Differential diagnosis of FPIES	
Infectious gastroenteritis	
Sepsis	
Necrotizing enterocolitis	
Inborn errors of metabolism	
Food adversion	
Neurologic disorders	
Anaphylaxis	
Gastrointestinal reflux disease	
Lactose intolerance	
Hirschsptrung disease	
Eosinophilic gastroenteropathies	
Celiac disease	
Immune enteropathies	
Obstructive problems	
Coagulation defects	
Alfa-1 Antitrypsin deficiency	
Primary immunodeficiencies	

The treatment of FPIES is based on the patient’s support with intravenous fluids and the avoidance of the trigger food. In case of reaction to cow’s milk, soy avoidance is suggested as well, because around 50% of the patients react also to it; therefore, the amino acid-based formula is used to replace both of them [18]. FPIES is a self-limiting syndrome, and its natural history shows tolerance to the culprit food at variable ages, depending on the type of food trigger and country of origin.

## Case presentation

### First clinical case

A two-month-old, exclusively breastfed male infant presented to the Emergency Department after two episodes of vomit and five episodes of diarrhea without blood or mucus. In the previous week, the baby had some occasional episodes of loose stools and a mild, but persistent, abdominal distension. He was born at term (39 weeks of gestational age) from spontaneous vaginal birth after an uncomplicated pregnancy, with a birth weight of 3480 g. Before he came to our attention, the boy was always in good health and his weight was regularly increasing. On physical examination, the child appeared pale and irritable, with low-grade fever (37.8 C°), heart rate 178 beats per minute and a prolonged capillary refill time (3 s). An intravenous bolus of 0.9% saline at 20 ml/Kg was administered in 20 min, followed by continuous intravenous hydration. Blood tests were in range, except for an increased C-reactive protein (3.5 mg/dl) and hypoalbuminemia (2.7 g/dl). The arterial blood gas analysis (ABG) was normal, except for a significant increase in methemoglobin (13%, normal range between 0 and 1.5%). Two urinary dipsticks in two different samples showed no signs of infection, later confirmed by a negative urine culture. Six hours after the admission, the child became irritable and his skin was mottled. Blood cultures were obtained before starting an intravenous antibiotic therapy with ceftriaxone. Central nervous system, thoracic and abdominal ultrasounds were negative. Eventually, the blood cultures and stool analysis (for bacteria, viruses, fungi and parasites) tested negative as well. The day after, through fluid resuscitation and antibiotic therapy, the boy’s conditions generally improved, and his mother kept breastfeeding him. However, he continued to present numerous episodes of diarrhea, around 12–15 per day, with greenish liquid stools, that led to a mild weight loss (− 200 g in 48 h). Two days after the admission, despite intravenous support, the child developed a severe dehydration, with hypernatremia (152 mmol/L), hypokalemia and hyperchloremic metabolic acidosis (pH 7.26, bicarbonate 18 mmol/L, base excess − 7.7, acid lactate 2.39 mmol/l). Blood tests showed anemia (hemoglobin 9 g/dl), thrombocytosis (platelets 552.000/mL), worsened hypoalbuminemia (1.7 g/dL) which required an IV supplementation, stably elevated methemoglobin (13%) and raised levels of transaminases (peak values: ALT 396 U/L, AST 121 U/L). Due to the worsening clinical condition and to a very poor suction, a central venous line was placed and parenteral nutrition was started. Remarkably, when breastfeeding was suspended because of the baby’s hypotonic suction, diarrhea started to improve and halved to five-six episodes per day. Serologies and PCR for cytomegalovirus, Parvovirus B19, Epstein Barr virus, Adenovirus, hepatotropic viruses, and a nasal-pharyngeal swab for viruses and bacteria were negative. In the following days, the patient regained good general clinical conditions, and laboratory tests were normalized, except for the methemoglobin, which was still very high (8–10%). However, when breastfeeding was reintroduced, his diarrhea worsened once again to 10–15 episodes per day. A metabolic disease was suspected but it was unlikely, as demonstrated by the negative neonatal screening, the clinical presentation and the values of ammonium, glucose, lactate and ABG, all in range. Congenital causes of diarrhea were ruled out because they should have appeared earlier, in the first days of life. Thus, according to some similar clinical cases reported in the literature, the close relationship between breastfeeding and diarrhea and the high level of methemoglobin were emphasized and Food Protein-Induced Enterocolitis Syndrome (FPIES) caused by maternal milk was suspected. Since the mother was already on a milk exclusion diet, breastfeeding was definitely stopped and replaced with an amino acid-based formula. Over the next 72 h, diarrhea gradually improved and then stopped, so that the intravenous hydration was reduced and subsequently suspended, and the infant started to gain weight. Skin prick test resulted negative for cow’s milk proteins (lactalbumin, lactoglobulin, and casein) and soy. After the hospital discharge, an oral food challenge test (OFC) was proposed to the child’s parents but they refused it, in consideration of the severe clinical symptoms that the infant previously developed. The boy was weekly checked through the following months and he kept gaining weight without presenting any clinical symptom. Therefore, the amino acid-base formula was continued, which he’s still taking at the age of 6 months.

### Second clinical case

A three-month-old, exclusively breastfed female baby presented to the Emergency Department for a 3 days history of persistent vomit and diarrhea without fever. She was born at term (39 weeks of gestational age) from spontaneous vaginal birth after an uncomplicated pregnancy, with a birth weight of 3210 g. Her previous medical history was silent and her growth has always been regular. On physical examination, the baby was pale and hypotonic and a weight loss of 6% was noticed. Blood tests showed mild anemia (hemoglobin 10.9 g/dL), lymphomonocytic leukocytosis (white blood cells 14.050/mmc, lymphocytes 7.370/mmc, monocytes 2.680/mmc), thrombocytosis (platelets 927.000/mmc), electrolyte imbalance (sodium 131 mEq/L, potassium 3.27 mEq/L, chloride 109 mEq/L, calcium 8.33 mg/dL), hypoproteinemia (3.2 g/dL) with hypoalbuminemia (1.8 g/dL) and slightly altered coagulation values (INR 1.6, aPTTr 1.85). Arterial blood gas was normal, except for a raised level of methemoglobin (7%, normal range between 0 and 1.5%). A venous catheter was placed and continuous intravenous hydration with 0.9% saline was started. Stool analysis tested negative for viruses, bacteria and parasites. Suspecting a post-viral intestinal colonization, an antibiotic therapy with rifaximin was started, later substituted by oral gentamicin, both without clinical improvement. Due to the persistent diarrhea and the alteration of blood tests, we integrated the girl’s therapy with vitamins, trace elements (such as zinc) and albumin, and we performed a red cells and a plasma transfusion. Even if she continued to assume breast milk regularly, we added supportive parenteral nutrition. Laboratory tests were requested to rule out myocarditis, metabolic diseases, botulinum intoxication, cystic fibrosis and pancreatic insufficiency, and they were all normal. The patient also developed a severe hypotonia with hypoexcitability of deep tendon reflexes. A cerebral MRI and an electromyography showed no remarkable alterations, and vitamin B12 and folate were in range as well. Because of the persistence of the intestinal symptoms, an esophagogastroduodenoscopy was requested. The histopathology of the biopsies showed an eosinophilic infiltration of the duodenal mucosa, raising the suspicion of a non-IgE mediated food allergy to cow’s milk proteins, conveyed by maternal breast milk. Therefore, the baby’s mother started an exclusion diet and stopped the consumption of cow’s milk and dairy products. In the following 2 days, we noticed a slow but steady improvement of the general conditions of the patient, which was still mildly hypotonic but showed reduced episodes of diarrhea. Therefore, an amino acid-based formula was started, leading to a rapid and stable regularization of the bowel function, a gradual increase in weight and a complete recovery of the muscle tone. Blood tests confirmed the clinical improvement with an increase in hemoglobin and total proteins and the normalization of coagulation parameters and methemoglobin. RAST and skin prick test for cow’s milk proteins tested negative. An oral food challenge test (OFC) was proposed to the child’s parents after the hospital discharge, but they preferred to not perform it. Therefore, the girl was kept on amino acid-base formula and her mother continued the exclusion diet. We monthly checked the infant for the following 9 months and she kept gaining weight, without presenting any clinical symptom. Weaning was regularly started at 6 months, with the exclusion of cow’s and soy milk and dairy products, which we’ll try to introduce in the next months.

## Discussion and conclusions

In order to identify and collect all the clinical cases of FPIES in exclusively breastfed children, we searched the medical literature on Pubmed, using different combinations of the following keywords: FPIES, food protein-induced enterocolitis syndrome, breastfeeding, breast milk, maternal milk. All the manuscripts have been analyzed with descriptive statistics according to several different variables, all reported in the following table (Table 4A and B).

**Table 4:** All the cases of FPIES in exclusively breastfed infants reported in the medical literature

**A**				
**Author**	**Age**	**First Diagnosis**	**Clinical Phenotype**	**Trigger Food**
Miceli Sopo [18]	1 months	Gastroenteritis	Chronic	Cow’s milk
Miceli Sopo [18]	2 months	\	Acute on chronic	Cow’s milk
Monti [19]	1 months	Gastroenteritis	Acute on chronic	Cow’s milk
Ntoumpara [20]	4 months	Gastroenteritis, Sepsis	Acute on chronic	Unknown
Vergara Perez [21]	2 months	Sepsis	Acute	Cow’s milk
Kaya [22]	15 days	Sepsis	Acute on chronic	Cow’s milk
Kaya [22]	2 months	\	Acute on chronic	Cow’s milk
Tan [23]	6 months	\	Acute	Soy
**B**				
**Author**	**Prick Test/RAST**	**OFC**	**T.F. Susp.**	**AAF**
Miceli Sopo [18]	Negative	Not performed	Yes	No
Miceli Sopo [18]	Negative	Not performed	Yes	No
Monti [19]	Negative	Not performed	Yes	Yes
Ntoumpara [20]	Negative	Non performed	Yes^a^	Yes
Vergara Perez [21]	Negative	Non performed	Yes	No
Kaya [22]	Negative	Positive	Yes	Yes
Kaya [22]	Negative	Positive	Yes	Yes
Tan [23]	Negative	Non performed	Yes	No

*T. F. Suspension* Trigger Food Suspension, *A. A. F.* Amino Acid-based formula

\ = No mention of a former diagnosis. ^a^ Although the trigger food was unknown, the patient’s mother started an exclusion diet without cow’s milk, dairy products and soy

Six articles were found, for a total of 8 patients. The age of onset varies from 15 days of life to 6 months of age, with an average of 3 months. This is consistent with the already known features of FPIES, as it can manifest from the first days of life for up to 2 years. In terms of clinical phenotype, most of these cases (62.5%) are characterized by an acute on chronic scenario. It means that these patients already presented some signs of an ongoing, chronic disease (watery stools, sporadic vomits, abdominal distension, poor growth) before developing an acute event (septic-like appearance, profuse vomit or diarrhea, severe dehydration) that led to an hospital admission. Two patients presented only acute symptoms and just one was affected by a purely chronic form of FPIES.

In terms of differential diagnosis, three of these cases were initially considered as a form of gastroenteritis, and three other times a form of sepsis was suspected. As for the food triggers, cow’s milk was the most frequently involved (75%), while soy is indicated as a causative factor only in one patient. In one case the trigger food was not identified.

In all the cases reported, the infants’ mothers started an exclusion diet. In 50% of the patients an amino acid-based formula was started, both to support the children nutritionally and to consolidate the diagnosis of FPIES. In all the cases, none of the patients presented a positive prick test or RAST test. An oral food challenge was performed in only two patients, in which it turned out to be positive. This is a remarkable finding, since a diagnosis of FPIES needs a positive OFC test to be formalized, otherwise it would be just presumptive. However, as we reported in our clinical cases, this test might not be accepted by the patients’ families who, understandably, don’t want to expose their babies to the same severe symptoms that led them to the hospital admission.

Finally, methemoglobin was not mentioned in any of the analyzed reports, albeit being a useful and simple tool to identify a case of FPIES [24–26]. Methemoglobinemia (i.e. pathological levels of methemoglobin) can be congenital or acquired. Congenital methemoglobinemia is caused by an enzymatic deficiency (typically involving the cytochrome b5 reductase) or by the presence of hemoglobin M, and it is clinically characterized by early-onset cyanosis (i.e. “blue babies”) [27]. Acquired methemoglobinemia is typically determined by either drugs or toxic chemicals, such as topic anesthetics (benzocaine, lidocaine), or by FPIES [28]. Both congenital conditions and drug assumptions can be excluded with a thorough anamnesis. Therefore, in an infant with gastrointestinal symptoms (vomit, diarrhea), poor growth and methemoglobinemia, FPIES should promptly be suspected [29].

The treatment is based on mother’s exclusion diet if a trigger food is identified. However, an amino acid-based formula should be considered as well, to nutritionally support the child, reduce the severity of the symptoms and speed up the recovery [30, 31].

In conclusion, FPIES in exclusively breastfed infants is a rare condition, which can be easily misdiagnosed. In the presence of compatible history and clinical symptoms, this diagnosis should be considered also in exclusively breastfed babies, as proteins that trigger this condition may be present in breast milk in sufficient quantities to cause it. A prompt improvement of the symptoms with amino acid-based formula should be seen as an important criterion to pragmatically diagnose FPIES. Methemoglobin is a helpful and rapidly available parameter that can simplify the diagnostic process and early detect this condition.

## Data Availability

The dataset supporting the conclusions of this article is included within the article (and its additional files).

## Abbreviations

FPIES: Food-Protein Induced Enterocolitis Syndrome
RAST: Radioallergosorbent test
OFC: Oral Food Challenge
ABG: Arterial blood gas analysis
IV: Intravenous
ALT: Alanine transaminase
AST: Aspartate transaminase
INR: International normalized ratio of prothrombin time
aPTTr: Activated partial thromboplastin time ratio
MRI: Magnetic resonance imaging
